# CRISPR/Cas: Advances, Limitations, and Applications for Precision Cancer Research

**DOI:** 10.3389/fmed.2021.649896

**Published:** 2021-03-03

**Authors:** Yue Yang, Jin Xu, Shuyu Ge, Liqin Lai

**Affiliations:** ^1^Department of Pathology, Tongde Hospital of Zhejiang Province, Hangzhou, China; ^2^Department of Otolaryngology, Tongde Hospital of Zhejiang Province, Hangzhou, China; ^3^Department of Pharmacy, Tongde Hospital of Zhejiang Province, Hangzhou, China

**Keywords:** clustered regularly interspaced short palindromic repeats, CRiSPR/Cas, cancer, precise cancer treatment, genetic editing, diagnosis, precision medicine

## Abstract

Cancer is one of the most leading causes of mortalities worldwide. It is caused by the accumulation of genetic and epigenetic alterations in 2 types of genes: tumor suppressor genes (TSGs) and proto-oncogenes. In recent years, development of the clustered regularly interspaced short palindromic repeats (CRISPR) technology has revolutionized genome engineering for different cancer research ranging for research ranging from fundamental science to translational medicine and precise cancer treatment. The CRISPR/CRISPR associated proteins (CRISPR/Cas) are prokaryote-derived genome editing systems that have enabled researchers to detect, image, manipulate and annotate specific DNA and RNA sequences in various types of living cells. The CRISPR/Cas systems have significant contributions to discovery of proto-oncogenes and TSGs, tumor cell epigenome normalization, targeted delivery, identification of drug resistance mechanisms, development of high-throughput genetic screening, tumor models establishment, and cancer immunotherapy and gene therapy in clinics. Robust technical improvements in CRISPR/Cas systems have shown a considerable degree of efficacy, specificity, and flexibility to target the specific locus in the genome for the desired applications. Recent developments in CRISPRs technology offers a significant hope of medical cure against cancer and other deadly diseases. Despite significant improvements in this field, several technical challenges need to be addressed, such as off-target activity, insufficient indel or low homology-directed repair (HDR) efficiency, *in vivo* delivery of the Cas system components, and immune responses. This study aims to overview the recent technological advancements, preclinical and perspectives on clinical applications of CRISPR along with their advantages and limitations. Moreover, the potential applications of CRISPR/Cas in precise cancer tumor research, genetic, and other precise cancer treatments discussed.

## Introduction

Cancer is one of the main causes of disease-associated mortalities worldwide with ever-increasing incidence worldwide (1). Comprehensive and large-scale sequencing databases have shown that genetic alterations, either specific to a certain type or common to several types, play crucial roles in tumorigenesis (2). Determining the structural and functional features of mutated genes, particularly long-tail molecular alterations, in genetic variations of cancer genomes play pivotal role in advancing cancer research (3, 4). However, systematic functional analysis of genes and mutations are time-consuming, expensive and laborious (5). Discovery of mutations that cause phenotypes relied either on random mutagenesis or indirectly on perturbation of transcripts by RNAi. The development of engineered nucleases such as zinc finger nucleases or transcription activator-like effector nucleases (TALENs) have made it possible to directly target and modify the genomic sequence (6, 7). Recently, genome engineering was greatly accelerated by the development of clustered regularly interspaced short palindromic repeats (CRISPR) technologies. Since the first use of CRISPR/CRISPR associated proteins (CRISPR/Cas) as a genome editing tool in 2013 in mammalian cells (8, 9), this toolbox has been extensively and continuously expanded. CRISPR/Cas systems are currently capable of not only manipulating the genomic sequence of cells and organisms, but also the introducing and site-specific targeting of epigenetic and transcriptional modifications (10–12).

In the past decade, the emergence of the CRISPR technology has brought revolutionary advances into genome engineering and made it powerful tool in different cancer researches including fundamental sciences to translational medicine and precise cancer treatment. The CRISPR/Cas are prokaryote-derived genome editing systems that have shown promising contributions to detect, image, manipulate and annotate specific DNA and RNA sequences in various types of living cells. CRISPR/Cas, capable of specific genome modifications in living eukaryotic cells, making this technology one of the key scientific discoveries of the twenty-first century. The genomic modifications include; sequence deletions, insertions, substitutions, integrations, and epigenetic genes regulation. In the last few years, advancements in this technology make an ability to drive into both basic and clinical research applications. CRISPR/Cas system is an RNA-guided targeted genome engineering platform, attaining a considerable attention in experimental research, and revolutionize different fields of life sciences. Functionally, the CRISPR-Cas system is divided into 2 classes according to the structural composition of the effector genes. The class 1 CRISPR system consists of multi-subunits of effector nuclease complexes and includes the type I, III, and IV CRISPR systems. The class 2 consists of a single effector nuclease, and routine practice of genome editing has been achieved by the development of the Class 2 CRISPR-Cas system, which includes the type II, V, and VI CRISPR-Cas systems. Types II and V are utilized for DNA editing, and type VI for RNA editing. CRISPR techniques can induce both quantitative and qualitative changes in gene expression through the DSB repair pathway, transposase-dependent DNA integration, base editing, and gene regulation using the CRISPR-dCas or type VI CRISPR system.

The CRISPR systems were first observed in *E. coli* in 1987 (13) and then in several other bacteria species (14). The exact functions and roles of these short repeat sequences remained unclear until in 2005, when strong evidences have hypothesized that these repeated sequences function as a part of an adaptive immune system in bacteria. Several studies have reported the similarities between the phage DNA and these repeated sequences (15–17). Further preclinical and animal model studies have demonstrated that CRISPR and CRISPR/Cas are associated to the adaptive immunity targeting foreign viral DNA (18). Mechanistically, two distinct RNAs including the CRISPR RNA (crRNA) and the trans-activating crRNA (tracrRNA) activate and guide Cas proteins to bind viral DNA sequences, which are subsequently cleaved together. The tracrRNA is a distinct type of RNA that interacts with the crRNA to produce the dual guide (g) RNA in CRISPR-Cas systems. The tracrRNA-crRNA interaction is pivotal for pre-crRNA processing, target recognition, and also cleavage.

CRISPR/Cas systems are adaptive (acquired) immune systems of prokaryotic and archaeal microorganisms and rely on ribonucleoprotein effector complexes. They eliminate invading phages, conjugative plasmids, and mobile genetic elements via reserving the memory of the encounters with foreign DNA in unique spacer sequences into CRISPR arrays (17–19). Naturally, CRISPR systems integrate foreign DNA molecule into CRISPR arrays, which subsequently produce crRNAs, and containing protospacer regions that are complementary to antigenic invading DNA molecules, followed by hybridizing each crRNA with other non-coding tracrRNA. A crucial event, which forms a hybrid of crRNA-tracrRNA, and makes a complex with Cas nucleases that cleave target-DNA sequences nearby to short sequences called protospacer adjacent motifs (PAMs) (20, 21). Genetic engineers can manipulate the CRISPR/Cas system efficiently and can target genes of interest to regulate their functions effectively in any eukaryotic organism, particularly in mammalian. The molecular biology of the CRISPR/Cas reveals how it can be operated while using synthetic guide RNAs (gRNAs) and other components to the target region of interest in DNA molecule for the desired application and finds the disease-causing genetic variations (22). Typically, the most widely used CRISPR system i.e., CRISPR/Cas9 targets 5′ of a PAM sequence. They induce double-stranded breaks (DSBs), which can be repaired by 2 DNA repair pathways called, homology directed repair (HDR) and non-homologous end joining (NHEJ) (22). HDR pathway facilitates precise gene modifications in the presence of a repair template (23). However, in the absence of a repair template, DSBs repaired by the NHEJ pathway that introduces insertion or deletions by editing DNA region, resulting in target genes disruption by shifting the reading frame (23, 24).

CRISPR/Cas nucleases-induced DSBs are mostly repaired by efficient eukaryotic cellular NHEJ pathway rather than by the HDR (25). Meanwhile, utilizing the Cas9 nickases can optimize the yields of indel at the genes loci, and enhance the HDR efficiency (26). The efficacy of the HDR pathway can be improved by enhancing the HDR pathway via gene silencing or suppressing non-homologous end-joining proteins activity (23), using small-molecule reagents (23, 27), or expressed proteins (26–28). Currently, DNA repair proteins have shown promising capacities in this regard, but *in vivo* implementation of these strategies are challenging. Moreover, DSBs in cells via DNA repair pathways are described that lead to many undesired genomic alterations, such as large deletions and translocations (29, 30). Various efforts have been made to improve HDR, such as DNA donor template designing, system delivery, and cell cycle synchronization (26, 31–33).

## Pros and Cons of CRISPR/Cas Technologies

In the last few years, advances in CRISPR/Cas technologies are spectacular and have shown considerable potential in several fields of life sciences research. CRISPR technologies are now considered more accurate, target-specific, easy to use, and multi-potential. Despite the remarkable advances in CRISPR, several limitations and concerns still exist, which need to be addressed and solved for the optimized Cas systems development. The current attempts at addressing all those concerns have been made to overcome these technical hurdles. In the following sections, the main limitations of the CRISPR technologies and recent advances to address them are discussed.

The off-target effects are still a major concern in complex eukaryotic organisms, most often *in vivo* for therapeutic applications (34, 35). The targeting specificity depends upon the gRNA of Cas9 and PAM sequences, and off-target cleavage in the genome (36). Different online editing programs have been developed and successfully utilized to identify and predict off-target cleavages *in silico*. However, these tools are limited to examining homologous genes and face shortcomings to predict, for example, epigenetic modifications. Technical advances like high throughput genome-wide next-generation sequencing, play an important role in reducing off-target effects (35, 37). Developing a well-optimized and engineered CRISPR system can significantly reduce the off-target effects. For instance, off-target effects can be reduced via increasing the nucleases cleavage specificity or reducing the time frame of functional activity for their applications. Different Cas proteins that exhibit enhancements in on-target specificity have been engineered that include eSpCas9, HF-Cas9, HypaCas9, and Sniper Cas9 (38–41). Another approach is using Cas9 nickases, where one of the endonuclease domains were catalytically inactivated and as a result, the low off-target effect was analyzed in the genome (42, 43). Off-target effects induced by CRISPR can be reduced by limiting the duration of Cas9 activity. For example, the Cas9 system delivered via electroporation had shown a shorter half-life than delivered by other vector systems such as lentiviral or plasmid vector system-based cargo delivery methods. Dosage affects several parameters and the target specificity of cleavage can play an important role in their applications. Alternatively, the target specificity of Cas9 systems can be enhanced by direct modulation of the activity of the genome-editing proteins, Cas9 proteins, by reducing their activity following the target locus alteration (44). The Cas9 nucleases were activated by inserting a modified 4-hydroxytamoxifen-responsive intein, a cell-permeable small molecule, at specific positions in Cas9 (44). These conditionally active Cas9 systems could alter the target genomic sites and were reported to enhance the target specificity human cells, up to 25-folds higher than the wild-type Cas9 (44–47).

Recent evidences have demonstrated that CRISPR system could be a highly efficient approach for the gene editing and manipulation applications in a variety of eukaryotic cells. However, HDR and indel mutation in some genome sites have shown low efficiency. To address the insufficient indel of Cas-system in the target sites, some efforts have been made to increase efficacy by either Cas engineering or gRNA (48, 49). The CRISPR/Cas proteins preceded DSB after the recognition of a PAM sequence (50, 51). Each type of Cas proteins contain their PAM sequence in the genome. Broadly speaking, type II CRISPR/Cas recognizes 3′ G-rich DNA sequences, while another type V, preferred 5′ T-rich sequences for their application.

The main issue in genome editing approaches is the unavailability of PAM in the desired gene loci. However, a range of Cas-nucleases variances such as SpCas9 and Cas12a are now available that are decreasing PAM restriction (52, 53). These kinds of advancements will provide flexibility in genome editing for the desired specific targets. In other ways, artificial intelligence plays a critical role and has been adopted for experimental designing to predict target sequences with high indel efficiency (54). The desired HDR efficiency to make genes functionally correct remains low, though different chemical and engineering tools have been used, i.e., chemical reagents, such as SCR7, NU7441, and KU0060648 (55, 56). The use of a donor template in the form of ssDNA led to increased HDR efficiency in cells (57). CRISPR/Cas often triggers cell apoptosis due to DSBs, rather than the desired genome editing (58). The safety issue raises when this genome editing system is utilized in human pluripotent stem cells (hPSCs). In response to DSBs by CRISPR, the activation of p53 occurred that triggers cellular apoptosis (59).

Recently, genome editing with base editors makes it possible to precisely fix desired targeted point mutations without requiring donor DNA templates, DSBs, or independence on HDR. In recent decades, these editing systems have been catalytically impaired nucleases, as a result, DSBs have not occurred. Importantly, 2 classes of base editors have been established; namely, cytosine base editors (CBEs) and adenine base editors (ABEs); they enable to catalyze the C•G base pairs (bp) conversion to T•A bp, and A•T bp to G•C bp, respectively (60–62). Besides these, catalytically inactivated CRISPR-dCas9 (dCas9) was applied for epigenome modifications instead of a genome that can alter gene regulation. CRISPRa and CRISPRi system has been developed to activate and silence genes, respectively (63). For example, dCas9 in combination with histone deacetylase (HDAC), improved CRISPR system efficacy and optimal positioning and developed an organized system to study epigenome (64). The evaluation of off-target effects can be analyzed through several online bioinformatics tools to predict potential off-targets with similar sequences, such as CCTop (https://crispr.cos.uniheidelberg.de), and Cas-OFFinder.

In addition, technical limitations and advances in the field of CRISPR technologies raise concerns for immunogenic toxicity. Recently, a study has shown that human subjects included, possessed pre-existing antibodies against Cas9. The obtained results showed that more than 50% of their subjects included in the study had immunity against the commonly used bacterial nucleases (65). In their study, the two extensively studied nucleases for gene therapy of Cas orthologs i.e., SaCas9 and SpCas9, were prevalent in human blood, and the human immune system has shown an immunogenic response against these nucleases. In this regard, extensive studies should be conducted particularly, for *in vivo* gene therapy applications. Furthermore, the gRNA triggers an innate immune response in human cells due to the presence of the phosphate group at the 5′ terminal (66). In addition, CRISPR has been extensively applied in clinical trials to modify somatic cells *ex vivo*, with the aim of reducing risk, and subsequently, transferring for *in vivo* gene therapy applications. However, the germ-line gene editing studies for therapeutic purposes still face ethical challenges. In this regard, the ongoing and near-future clinical trials on somatic CRISPR therapy need to be evaluated for the long-term to check the system efficacy and safety.

## CRISPR Delivery Approaches and Challenges

An efficient delivery of both Cas9 and the single guide RNA (sgRNA) to the target cell is required for a successful *in vivo* administration of CRISPR/Cas9. The delivery approach should have high editing efficiency, induce low immunogenicity and deliver the Cas9/sgRNA specifically to the target organ or cell type. The first generation genome editing strategies in mammalian cells have been utilized the plasmid based expression of Cas9 and sgRNA (8, 9). Moreover, this approach is efficient for *in vivo* applications in model organisms such as mice because the plasmid can be delivered to the tissue by hydrodynamic injection (67) or electroporation (68–70). However, in these applications the targeting delivery and editing efficiency are limited and control over the Cas9 activity is poor. Therefore, different viral and non-viral delivery strategies have been developed to enhance the performance of *in vivo* delivery of Cas9/sgRNA (71–73).

Adeno-associated virus (AAV) vectors are effective and among the most common used viral vectors for gene therapy because of their unique features including non-integrating nature, high transduction efficiency and serologically compatible with most of human population (74–77). Furthermore, the rich diversity of serotypes with distinct tissue tropisms enables AAVs to selectively target different organs (78, 79). However, the main limiting issue of AAVs for delivery of CRISPR and Cas9 is the limited cargo size of AAVs, so that the Cas systems and sgRNA should be encoded on additional separate vectors (74). AAVs can be administrated systemically or directly applied to the target organ for genome editing applications (75, 80–82). Using lipid nanoparticles (LNPs) is an alternative approach to viral delivery, which offer availability, low cost and high compatibility (83–85). LNPs have been employed for successful delivery of siRNA and mRNA in clinical trials (83, 86). Moreover, recent studies have demonstrated that LNPs can encapsulate and deliver the sgRNA and Cas9 mRNA to murine liver with high delivery efficiency and targeting performance (87–90). Furthermore, multifunction and modified nanoparticles can be additionally loaded with a donor template and thereby allow homology directed repair (91). However, the nanoparticles based carriers with a donor template suffer low editing efficiency (91). The main focus of the current research is on improving and establishing CRISRP/Cas9 as a gene repair tool. However, it is expected that CRISPR/Cas9 would be translated into a therapeutic agent for cancer treatment in clinical setting. To achieve this goal, the main step is developing effective carriers for tissue-specific delivery of Cas9/sgRNA (92–94).

Low editing efficiency in tumors and potential toxicity of the currently available delivery systems are the main limiting factors against translation of CRISPR/Cas9 technology into cancer therapeutics. The presence of an appropriate and effective alternative of delivery strategy is critical for CRISPR/Cas9 delivery, particularly where genome editing systems should be effectively conducted in the targeted organisms or cells. Until now, *in vivo* delivery of the Cas9 system remains challenging. Both physical techniques and viral vectors have been utilized for the delivery of the Cas9-based gene editing platform. The physical approaches are more feasible for *in vitro* delivery, but the viral vectors based techniques usually suffer limited packing capacities and poor safety profile. Recent preclinical and animal studies have demonstrated promising delivery performance and targeting efficacy of non-viral drug delivery systems such as polymeric and lipid nanocarriers for the delivery of CRISPR/Cas9 systems. These non-viral vectors are expected to be candidate carriers for the genome editing platform in the near future. The efforts in optimizing cationic nanocarriers with structural modification are described and promising non-viral vectors under clinical investigations are highlighted.

Different studies have recently developed a safe and effective strategy for antibody-targeted cell-specific delivery of mRNAs and siRNAs through systemically administration of LNPs (95–97). In this regard, few studies have reported promising outcomes in using LNPs for the delivery of Cas9 mRNA and sgRNAs. The initial findings showed that aminoionizable LNPs could serve as a safe and efficient carrier for Cas9 components (87). Rosenblum et al. reported a single intracerebral injection of CRISPR-LNPs against *PLK1* (sgPLK1-cLNPs) into metastatic orthotopic glioblastoma enhanced the *in vivo* gene editing specificity up to ~70%, which inhibited tumor growth by 50%, induced tumor cell apoptosis, and enhanced survival by 30% (87). The cLNPs were engineered for antibody-targeted cell specific delivery to reach the distributed tumors (87, 88, 97, 98). Intraperitoneal injections of sgPLK1-cLNPs targeting EGFR improved the site specificity of gene editing *in vivo* by 80% for distributed ovarian tumors, and inhibited tumor growth, and increased survival by 80% (87). The capacity of disrupting gene expression *in vivo* in tumors is a promising feature for translating CRISPR tools into clinical applications and paves the way for developing gene editing techniques for cancer research and treatment and potential applications for targeted gene editing of non-malignant tissues.

### Methods of Delivery

CRISPR technology has been reported one of the most promising therapeutic tool that could efficiently correct a variety of disease-associated mutations. In this view, it must be transported directly to their target site.

Multiple techniques have been developed for CRISPR delivery such as physical, viral, and non-viral delivery systems (99). Physical methods include microinjection, transfection, and electroporation that are most suitable for research purposes in cell culture. However, these strategies can be used for *ex-vivo* cell manipulation for adoptive transfer (100). Multiple studies revealed the delivery of Cas9 protein/gRNA ribonucleoprotein complexes into many cells of mammals by electroporation or transfection mediated by liposomes (101, 102). The findings of the studies have reported that the rate of insertion/deletion (InDel), induced by nuclease was 87% in induced pluripotent stem cells (iPSC). Furthermore, off-target cleavage was decreased, as compared with the transfection in plasmid DNA (102). Cas9/gRNAs delivered by lentiviral transduction or plasmid transfection have a longer half-life relative to Cas9–RNP complexes delivered through electroporation. Furthermore, Cas9-RNPs are active immediately post-delivery due to no lag, however, protein synthesis occurs. A reported study has been revealed that LNPs can efficiently deliver Cas9-RNP (71). Viral vectors, such as adenovirus, lentivirus, and adeno-associated virus (AAV) vectors have been used for delivery in clinical trials. Lentiviral vectors have been derived from HIV that provide stable and efficient delivery and can infect dividing as well as non-dividing cells, including the brain cells. Viral genes, such as vpr, vif, and nef are not needed for packaging. Therefore, the underlined genes are deleted while the expression of packaging genes are provided on separate plasmids to decrease the probabilities of reconstruction of wild-type virus (103). Moreover, lentiviral vectors are not suitable for therapeutic uses due to integration but this risk can be lowered via IDLV (104). Adenoviruses are viruses containing a linear double-stranded DNA genome of around 36 Kbp in length with four early and five late transcription units. The majority of the vectors are based upon adenovirus type 5 (Ad5). A recombinant virus has been constructed by removal of the early gene E1 or E1 plus E3 and grown in a packaging cell line that shows the expression of E1 to form infectious recombinant virus. Adenovirus can infect dividing as well as non-dividing cells and not show integration into the host genome (104).

**Figure 1:**
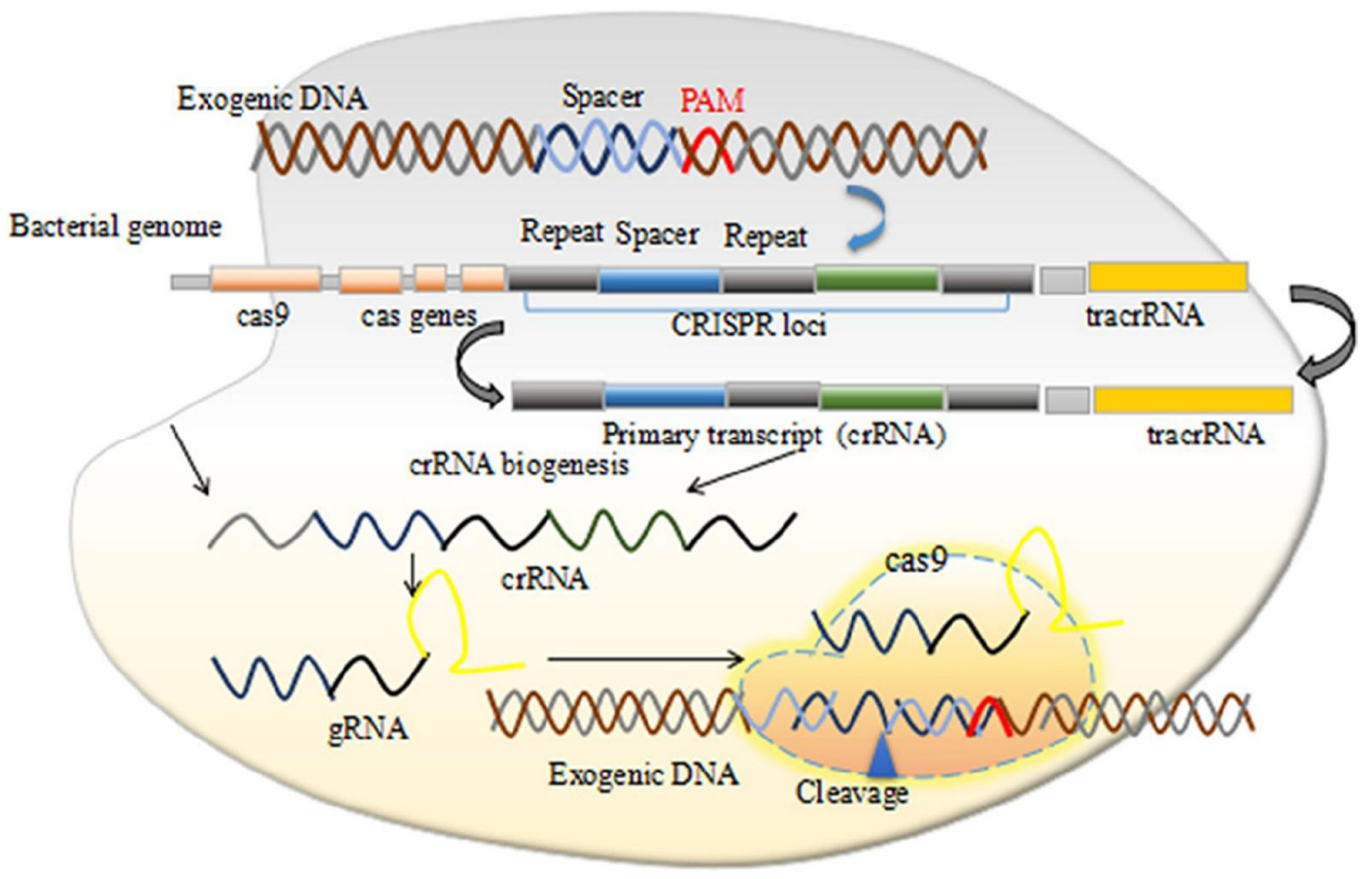
The CRISPR/Cas9 mechanism of action. Permission from (107).

Despite these applications, lentivirus and adenovirus vectors having some drawbacks, particularly safety problems associated with their immunogenicity (105). AAV vectors have significantly lower immunogenicity. AAV is a 4.7 Kb single-stranded DNA virus that needs E1 for the packaging of infectious viruses and can transduce dividing as well as non-dividing cells. In infected cells, the AAV genome can persist in an episomal form, but infrequently shows integration in the host genome. The most commonly used vectors for delivery of Cas9 are AAV because these vectors are very efficient and low immunogenic (106). However, the large size of the Cas9 endonuclease is a complication in its effective delivery with the gene for Streptococcus pyogenes Cas9 being about 4.2 Kb, while the size limit for AAV is between ~4.5 to 4.9Kb (Figure 1). Ran et al. (108) described Cas9 orthologs and revealed that Streptococcus aureus (SaCas9) shows similar potency of editing to SpCas9, but is over 1 Kb shorter and can specifically and efficiently perform gene editing. In recent years, the isolation of another Cas9 ortholog has been carried out from Campylobacter jejuni, which is shorter with a size of 2.95 Kb (109). It has been revealed that the packaging of the CjCas9 gene could be performed in an AAV vector along with gRNA and a marker gene for the generation of high viral titers that may deliver more specific CjCas9, and was revealed to be a targeted endonuclease.

## CRISPR Applications in Cancer Research

**Figure 2:**
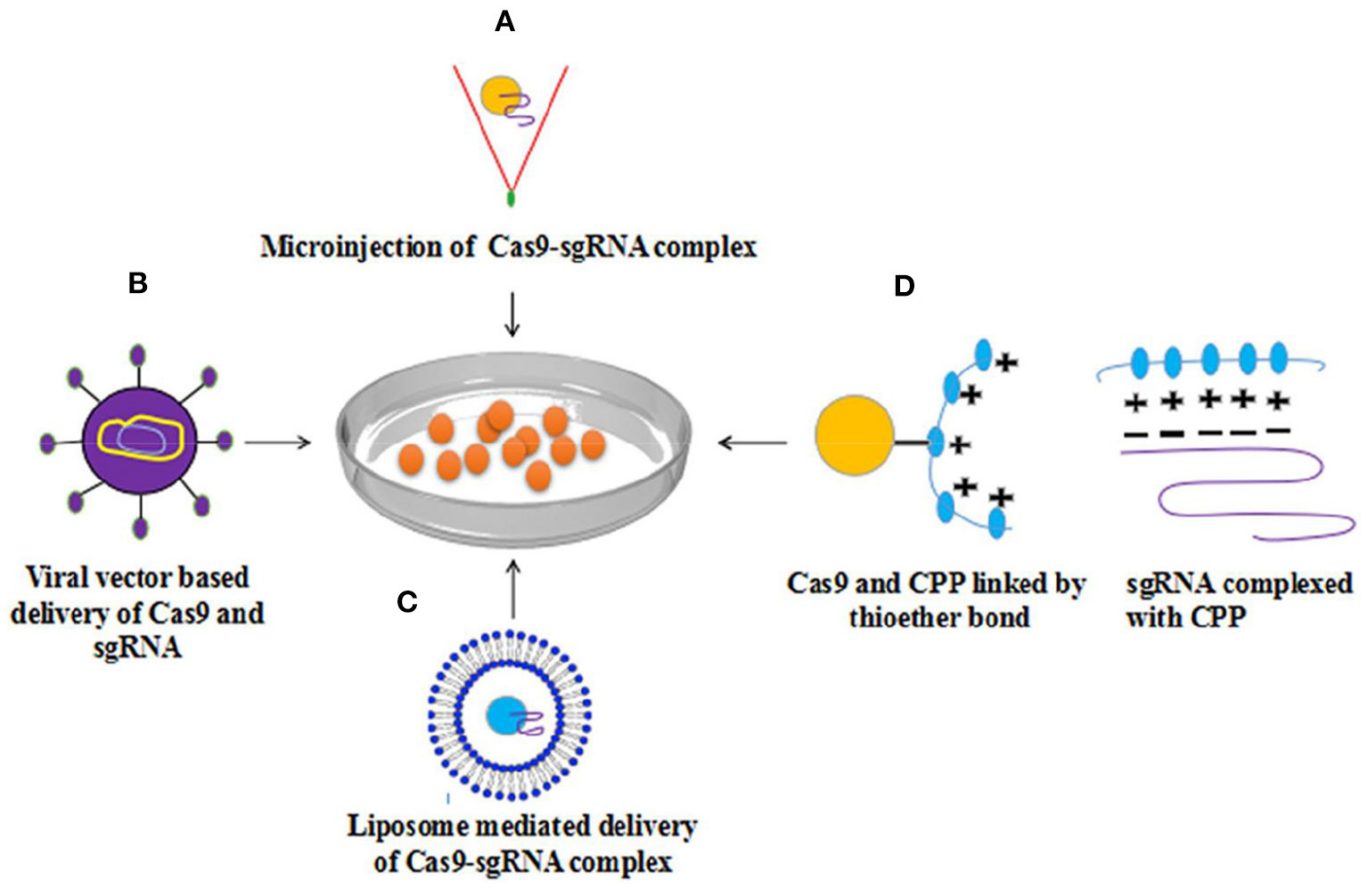
Methods for delivery of Cas9-sgRNA complex to cell **(A)** Microinjection based delivery of Cas9-sgRNA **(B)** viral vector (AAV) based delivery **(C)** Lipofection **(D)** Cas9-sgRNA complex delivery into mammalian cells via Cell-penetrating peptides (CPP) revealed considerable genome editing with elevated level efficiency. With permission (107).

Cancer is a disease of aberrant cell signaling that occurs due to a variety of genetic and epigenetic alterations in DNA. These alterations include the oncogenes, which enhance cell proliferation, and the tumor suppressors, which regulate cell growth and metabolism. The underline alterations lead to cancer progression. Nowadays, the ability of CRISPR to correct such cancer-associated alterations is an important objective for cancer diagnosis, cancer therapy, and other related applications. Hence, CRISPR is a promising tool that has been widely adopted in oncology research (Figure 2) with focusing on; animal tumor model construction, the discovery of new drug targets; cancer gene therapy, genetic screening related to drug resistance, and many others. In the below section, some of the promising applications of CRISPR in cancer research are summarized.

### CRISPR for Tumor Research Modeling

Understanding complex mechanisms at the molecular level that drive tumor progression is a crucial step to advance therapeutics development. Usually, tumors arise due to multiple gene mutations, and this complexity makes it difficult for the development of full-pledge cancer models. In this view, the CRISPR system was considerably used to establish rapid tumor models, both *in vitro* and *in vivo*. These models allow identifying the genetic determinants and a comprehensive detail of the mechanisms that underlying tumor occurrence, progression, and development.

**Figure 3:**
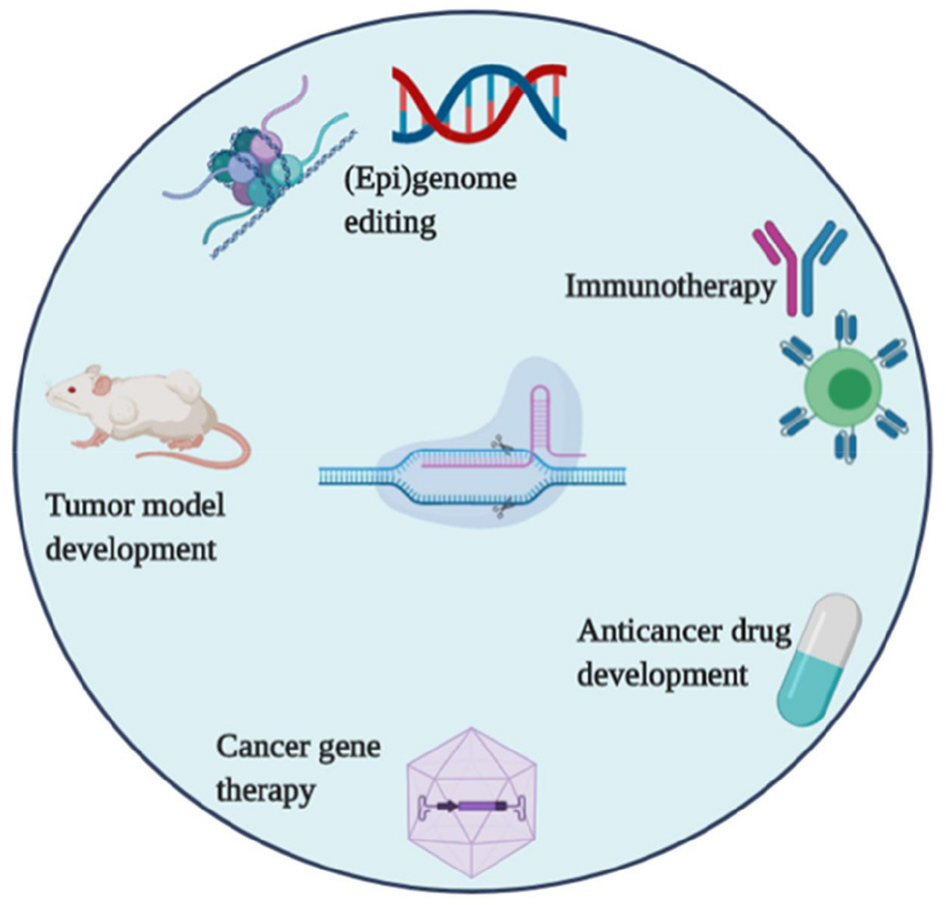
CRISPR/Cas systems applications in tumor research, drug development, and cancer therapies.

The generation of *in vitro* cancer model, while using CRISPR/Cas in mammalian cell lines with single or multiple gene(s) deletions is now easy and feasible (110), such as CRISPR-based mediated silencing of MELK, a cancer drug (OTS167) target in several clinical trials. The inactivation of MELK via CRISPR remains sensitive to OTS167 and does not affect the potency of cancer-derived cell lines. The underlined study explores the use of CRISPR that accelerate targeted cancer therapy research (111). Furthermore, CRISPR is applied to knock in or knock out functional alleles to develop drug resistance *in vitro*. CRISPR makes it possible to quickly evaluate candidate genes or specific mutations, associated with drug resistance (112). In this regard, a study was performed using CRISPR to identify mutations in crucial genes involved in therapeutic resistance that might be used for drug developmental strategies. For instance, NAMPT has been identified as the main drug target for the anti-cancer agent i.e., KPT-9274 (113). CRISPR, a versatile tool can be utilized to explore the genetic complexity of human cancer malignancies, such as myeloid malignancies, a malignancy that is driven by mutations in several genes, including Dnmt3a, Trp53, Tet2, Runx1, Ezh2, Smc3, Nf1, and Asxl1. By using the CRISPR system in single mouse hematopoietic stem cells, up to 5 genes were modified that induce the myeloid malignancies in mice (114). This study highlights the role of multiple gene mutations in cancer. CRISPR/Cas systems can be used to establish an *in vivo* tumor model. In exploring a complex mechanism of tumorigenesis, the *in vivo* cancer models play a critical role in the finding of key events i.e., pathogenesis and drug resistance. For example, the CRISPR system was used to attain mutations in important genes; P53, Kras, and Lkb1 in mice. These modifications/mutations in mice led to pathological changes in lung adenocarcinoma (115). The delivery of CRISPR cargoes into the living system plays an important role in model generation. For example, a lentiviral vector system used to deliver CRISPR into the desired target organs *in vivo* that create specific malignancy models (Figure 3) (116, 117).

### CRISPR-Based Screening Approaches in Cancer

CRISPR is effectively utilized to facilitate the discovery of next-generation targets or candidate genes that are sensitive or resistant to cancer therapy. Using the CRISPR system, several genetic screening studies were performed *in vitro*. It has been reported that in melanoma, the CRISPR library was applied to find the drug resistance mechanism of vemurafenib (PLX), an inhibitor of the BRAF protein kinase. In this study, new PLX-resistant candidates namely; TADA1, TADA2B, CUL3, and NF2 have been revealed (118). Moreover, using cell lines, the CRISPR screening has been utilized to identify oncogenes, and tumor suppressors *in vivo* (119, 120). The study showed the loss-of-function genetic screens and *in vivo* tumor mice models using CRISPR, and confirmed candidates gene and the pathways that are connected in the sensitivity and development of resistance to cancer immunotherapy (121). Strong evidences have highlighted the role of the Cas9 system in combination with RNA scaffolds that can be applied to induce site-specific epigenetic and transcriptional modifications while targeting a crucial region of the OCT4 promoters (122, 123). The OCT4 gene is recognized as one of the key players, which plays a critical role in tumorigenesis and therapy resistance.

### Targeting Gene Regulation in Cancer

In cancer, gene regulation has affected both post-transcriptional and translational modifications that evolve cancer cells to survive and adapt within the microenvironment. For example; the Knockdown of micro RNAs (miRNAs) that enhance tumor initiation and development can prevent tumor occurrence, development, and anti-cancer therapy resistance. In this view, a study found a knocked-out miR-17 in colorectal cell line and injected into nude mice (124). The obtained results showed a stable gene-phenotype even after 2 weeks in tumor tissue which indicated that CRISPR can play a critical role in targeting miRNAs and can effectively target tumorigenic miRNAs. In cancers, abnormal expression of epigenetic regulatory genes plays an important role in tumorigenesis processes. Targeting acetyltransferase p300 (associated with a catalytic histone H3 lysine acetylation) using CRISPR system can activate gene promoters and co-regulatory components, which in turn facilitate the expression of the target gene and the associated genes (125).

### Tumor Immuno-Regulation and Immunotherapy Approaches

Tumor immune escape is one of the key mechanisms of the cancer cell to survive and adapted in the tumor microenvironment, while the immune system unable to recognize it. Subsequently, tumor cells leash the immune cells through multiple pathways and thereby tumor cell progression and metastasis occur. Cancer immunotherapy is considered as an attractive strategy to target cancers and emerged as a potential therapeutic modality for the treatment of cancers. However, issues are existing to make it more precise for cancer patients (126, 127). In recent years, genetically engineered T cells against tumors have shown remarkable therapeutic effectiveness and performance. In human immune system, T cells play crucial roles in protecting the human body from infection by pathogens and eliminating mutant cells through specific recognition by T cell receptors (TCRs). Cancer immunotherapy utilizes the TCRs based recognition strategy to enhance the antitumor efficacy of T cells through releasing the inhibition of immune checkpoints and expanding adaptive immunity by promoting the adoptive transfer of genetically engineered T cells. T cells genetically equipped with chimeric antigen receptors (CARs) or TCRs have demonstrated significant effectiveness in treating different hematological disorders. However, the main issue of this approach is limited efficacy of engineered T cells in treating solid tumors. CRISPR system provides a new way to make engineered T-cells more efficient for the clinical treatment of different types of cancers (128). Moreover, the production of chimeric antigen receptor T (CAR-T) cells are significantly associated with the cancer therapy. Using CRISPR/Cas9, T-cells are genetically engineered *in vitro*, where the genes have been inserted and CAR protein have been expressed on the cell-surface that activated and recognized antigen on malignant cells very efficiently (129). Currently, several clinical trials are underway, using CRISPR for cancer immunotherapy applications (clinicaltrials.gov). However, several efficacy and safety challenges still exist on using CRISPR/Cas for clinical applications.

## Conclusion and Perspectives

The precipitous development in CRISPR technologies to their versatile and precise genome engineering in the last few years has been spectacular. These versatile tools now consider as an umbrella term, which revolutionized the life sciences and enabling advances in basic research for a variety of applications. It is believed that CRISPR can be established in clinics that can offer many therapeutic opportunities for treating human diseases, including cancer. Continued progress to improve and revolutionize new ways to deliver genome engineering tools into cells, and advance their capabilities to edit can implement these technologies for many therapeutic applications. CRISPR/Cas systems are widely utilized in tumor research for many applications both *in vitro* and *in vivo* models. Several clinical trials are currently underway, using the CRISPR/Cas system to accelerate or making the therapies more reliable to treat cancer effectively. However, extensive research work is still required to develop and applied these technologies in clinics. These technologies can provide wide-ranging opportunities for specific and desired genome engineering and can become a potent asset for the modern era of medicine. Continuous efforts to understand all their pitfalls, improving editing capabilities, and advances in the delivery systems will ensure the CRISPR system for the full potential to benefit society in near future.

## Author Contributions

YY and LL: conceptualization, methodology, and writing—review and editing. YY, JX, SG, and LL: resources, data curation, and writing—original draft preparation. YY: supervision, project administration. YY and JX: funding acquisition. All authors contributed to the article and approved the submitted version.

## Conflict of Interest

The authors declare that the research was conducted in the absence of any commercial or financial relationships that could be construed as a potential conflict of interest.
